# A Defect in *NIPAL4* Is Associated with Autosomal Recessive Congenital Ichthyosis in American Bulldogs

**DOI:** 10.1371/journal.pone.0170708

**Published:** 2017-01-25

**Authors:** Margret L. Casal, Ping Wang, Elizabeth A. Mauldin, Gloria Lin, Paula S. Henthorn

**Affiliations:** 1 Section of Medical Genetics, Department of Clinical Studies, School of Veterinary Medicine, University of Pennsylvania, Philadelphia, Pennsylvania, United States of America; 2 Department of Pathobiology, School of Veterinary Medicine, University of Pennsylvania, Philadelphia, Pennsylvania, United States of America; University of Sydney Faculty of Veterinary Science, AUSTRALIA

## Abstract

Autosomal recessive congenital ichthyosis in the American bulldog is characterized by generalized scaling and erythema with adherent scale on the glabrous skin. We had previously linked this disorder to *NIPAL4*, which encodes the protein ichthyin. Sequencing of *NIPAL4* revealed a homozygous single base deletion (CanFam3.1 canine reference genome sequence NC_06586.3 g.52737379del), the 157^th^ base (cytosine) in exon 6 of *NIPAL4* as the most likely causative variant in affected dogs. This frameshift deletion results in a premature stop codon producing a truncated and defective NIPAL4 (ichthyin) protein of 248 amino acids instead of the wild-type length of 404. Obligate carriers were confirmed to be heterozygous for this variant, and 150 clinically non-affected dogs of other breeds were homozygous for the wild-type gene. Among 800 American bulldogs tested, 34% of clinically healthy dogs were discovered to be heterozygous for the defective allele. More importantly, the development of this canine model of autosomal recessive congenital ichthyosis will provide insight into the development of new treatments across species.

## Introduction

Autosomal recessive congenital ichthyosis (ARCI, Online Mendelian Inheritance in Man [OMIM] #612281) is a rare skin disorder caused by defective formation of the skin barrier—the stratum corneum (SC). The hydrophobic SC regulates water movement into and out of the skin and protects the body from environmental, microbial, and chemical insults. The SC represents a unique evolutionary adaptation that allowed vertebrates to exist in a terrestrial environment. When defective, the clinical phenotype (e.g. scaling) reflects the body’s attempt to restore hydrophobicity and repair the flawed skin barrier. ARCIs are monogenetic disorders caused by variants in the genes that encode a wide array of molecules, including enzymes, structural proteins, and lipids, involved in the formation of the SC.

ARCI represents a group of rare inherited skin disorders characterized by excessive scaling. In humans, the incidence of congenital ichthyosis is 5–10 per 100,000 births in the United States [1]. This disorder usually presents at an early age and continues to affect the patients throughout their lifetime. Currently, there are seventeen genes associated with ARCI: *TGM1*, *ABCA12*, *ALOXE3*, *ALOX12B*, *ALOX15B*, *NIPAL2*, *NIPAL4*, *CYP4F22*, *PNPLA1*, *LIPN*, *NIPA1*, *NIPA2*, *CERS3*, *SLC27A4*, *SPINKS*, *LI5* and *KRT10* [2–4]. While the genetic causes have not yet been determined in all cases of human ARCI, a recent study demonstrated that *NIPAL4* disease-causing variants are equally prevalent with *ALOX12B* variants, followed by *TGM1* for most causative variants in Scandinavian patients [5]. The exact function of NIPAL4 is currently unknown but it functions as a magnesium transporter for fatty acid transport protein 4 (FATP4) and is associated with transglutaminase-1 and FATP4 in processing lipids to maintain epidermal barrier function [4].

**Fig 1:**
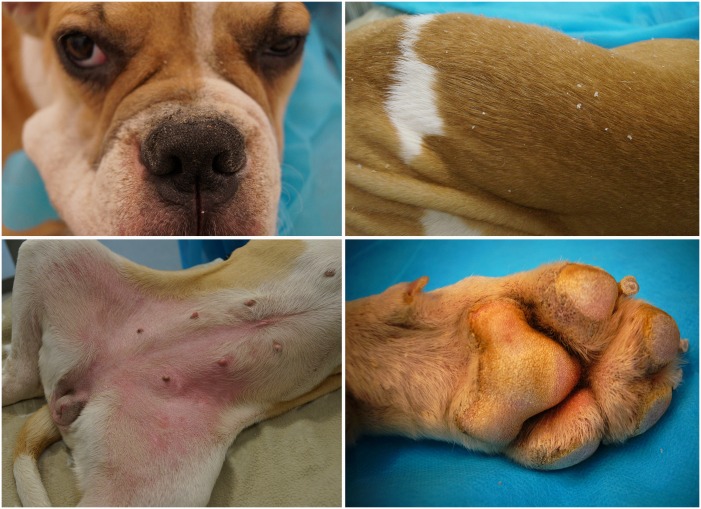
12-week-old, female, American bulldog with ARCI. Top left: erythema of the rostral muzzle with scaling. Top right: generalized soft white scale is present on the entire body. Bottom left: The abdomen is erythematous with tightly adherent light brown scale. Bottom right: Paw pad hyperkeratosis with yellowish discoloration.

We had previously described a form of autosomal recessive congenital ichthyosis in American Bulldogs (ABDs), including clinical presentation (Fig 1), mode of inheritance, and the histopathological features of this disorder [6]. The most significant findings of ARCI in these dogs were (1) the absence of ichthyin in the outer layer of the epidermis of ARCI-affected ABDs and (2) the identification of an ARCI-associated molecular marker, a short interspersed element insertion polymorphism near the *NIPAL4* gene. These findings suggested that a defect in *NIPAL4* was causative for ARCI in ABDs. We present here the discovery of a disease-associated variant in *NIPAL4* that establishes ARCI in ABDs as an animal model that can be used to better understand the disease process in humans and to develop therapies for this scaling disorder.

## Materials and Methods

### Animals and samples

Animal tissue samples (buccal swabs, EDTA blood, and skin biopsy samples) used in this study were recruited through veterinarians, breeders, and dog owners from the United States, Australia, Canada, and more than a dozen European countries. A clinical information questionnaire, a pedigree, and a signed informed consent document were obtained for each participant. The affected status of a dog with ARCI was verified by histopathological examination of skin biopsy samples by a board certified dermatopathologist (EAM) as previously described [6]. The study was approved by the Institutional Animal Care and Use Committee at the University of Pennsylvania (804197).

### DNA extraction, amplification and sequencing

Genomic DNA extraction, polymerase chain reaction (PCR) amplification, and Sanger gene sequencing were performed as previously described [6]. Briefly, blood and tissue extractions were performed using the QIAamp DNA MiniKit (Qiagen, Valencia, CA) according to the manufacturer’s protocol. PCR primers (Table 1) were designed to amplify six exons of *NIPAL4* gene from DNA obtained from an ARCI affected dog, and its clinically healthy parents and littermates. Primer design was based on sequences available through NCBI-Genbank (Gene ID: 489157 version Sep. 10, 2016, annotation release 104), http://www.ncbi.nlm.nih.gov, Ensembl (CanFam3.1, Gene ID: ENSCAFT00000027510), http://www.ensembl.org and UCSC (Dog May 2005, Broad/CanFam2, Assembly, http://genome.ucsc.edu). Amplification products were extracted from the gel using the QIAquick Gel Extraction Kit (Qiagen) protocol and the reagents supplied. After amplification and gel extraction, samples were submitted for sequencing to the DNA Sequencing Center (Abramson Cancer Center, University of Pennsylvania).

**Table 1:** PCR primers and conditions used in sequencing the canine *NIPAL4* gene.

Exon	Sequence(5’- 3’)	Tm (°C)	Extension (Second)	Amplicon (bp)
1	Forward: CCAGGACGCGGGGTGACTCReverse: TAGCGGTGGGGGTACGGGGTTTAG	64	30	717
2	Forward: CAAGGTGCCTGACCTTTCATReverse: GAAGGTCTCCTGTTGGTGGA	62	30	702
3	Forward: GCCTGTGAGAGATCCAGGAGReverse: CTGTGGAATGGGAAAACTGG	62	30	702
4	Forward: AAGAAGGCATCCTTCCACAAReverse: TCTCCAGCTCATGCTTCTCA	56	30	699
5	Forward: ACTTTGAGGAGGGGTGTGGTReverse: CTAAGGGTTGGGCTCTGAAGTC	63	30	1032
6–1	Forward: TCATGACCTAAATCACCTTCReverse: GTTCTGCCGCCACCACTCAC	62	45	1175
6–2	Forward: CCCCCGCTATGGACAAAGGAReverse: CTGGTGATGGGGTGCTGGAA	60	30	553
6–3	Forward: CAAGAACCCGGGTGGAAAGGGReverse: CCGCATCCCTCCAGAGATCA	62	30	773
Mutn	Forward: TTCAGAGCCCAACCCTTAGTGCReverse: AGATGGGGAACACCAAGGAGGTA	64	30	415

The sequencing results were compared to the published canine genome sequence (NCBI-Genbank, Gene ID 489157, CanFam3.1, annotation release 104, September 10, 2016, http://www.ncbi.nlm.nih.gov), as well as the simultaneously sequenced result of a clinically healthy ABD.

#### Comparison to human NIPAL4 gene and predicted protein

Based on the canine genome reference sequence (see above references), the canine *NIPAL4* gene contains 6 exons and encodes a 404 amino acid protein. Our analysis of the predicted protein differs from the Ensembl Gene ENSCAFG00000017367, and transcript pENSCAFT00000027510 predictions [7], which assumes a 2 bp deletion within exon 1 (and annotates it as two separate exons) and thus in the protein translation, and does not begin translation with a methionine residue. In addition, transcription start sites of the *NIPAL4* gene were inferred by examination of RNA sequencing information from the Broad Improved Canine Annotation v1 tracks [8] on the UCSC Genome Browser at http://genome.ucsc.edu, where no transcripts included the entire predicted amino terminus of the coding region predicted by Ensembl. Finally, there is no other methionine codon upstream of our predicted translation start site that is in frame.

Since there is no reliable canine *NIPAL4* reference cDNA and protein prediction, we refer to the predicted canine NIPAL4 gene protein product by comparison to the most common human transcript and protein (NM_001099287.1 and NP_001092757.1, respectively). Alignment of the human transcript 1 NIPAL4 protein sequence to our predicted canine NIPL4 protein sequence was performed using the Lipman-Pearson algorithm implementation in the DNASTAR (Madison, WI) Megalign sequence alignment software. The human protein is 63 amino acids longer at the amino terminus than our prediction of the canine NIPAL4 protein at the amino terminus due to translation starting from an upstream methionine residue not found in the canine (and other mammalian species) *NIPAL4* gene. Over the 404 amino acids of overlap between the predicted canine NIPAL4 polypeptide and the human NIPAL4 transcript 1 protein (RefSeq NP_001092757.1), the sequences align without gaps beginning at amino acid position 63 in the human protein, and with 365 out of 404 identical amino acids (90.3% protein sequence identity). All of the annotated protein sequence features of the human protein (NP_001092757.1), including nine predicted transmembrane regions each of 21 amino acids in length, are present in the 404 amino acid region of overlap. The sequences of seven of the transmembrane regions are identical between human and predicted canine *NIPAL4*, with transmembrane regions 5 and 6 each differing by two amino acids between human and predicted canine *NIPAL4*.

#### Restriction fragment length polymorphism (RFLP)

A PCR-RFLP based assay was developed for the ARCI-associated variant. The DNA fragment encompassing the variant in exon 6 was amplified using the PCR primers (Mutn forward and reverse; Table 1) to produce an amplicon of 415 bp. The restriction endonuclease HphI (10,000 U/ml, New England Biolabs, Ipswich, MA) was used as recommended by the manufacturer to digest 10 μl of the PCR product giving rise to fragments of 255 and 160 bp in the PCR product from normal dogs. PCR products were separated on 2% agarose gels by electrophoresis and visualized by staining with ethidium bromide. The cytosine deletion in exon 6 of *NIPAL4* destroys the HphI restriction site, resulting in an undigested 415 bp fragment.

#### Real-time PCR based allelic discrimination genotyping test

A real-time PCR based discrimination genotyping method for variant and wild-type alleles was developed (Applied Biosystems, Carlsbad, CA) and performed on 800 DNA samples from ABDs and 150 DNA samples from clinically healthy dogs of other breeds using previously established protocols [6] and the unlabeled PCR primers (forward: TTCTCCGTGTCTGCTGTCAAG; reverse: GGCATCCCCTGGAAGAAGTTC) and fluorescently labeled TaqMan^®^ 3′-Minor groove binder probes: VIC^®^ dye-labeled CTGGGCATCACCATAAA for wild type, and FAM^™^ dye-labeled CTGGGCATCACATAAA for the cytosine deletion mutant.

## Results

### Animals and variant discovery

All ARCI affected dogs were diagnosed by clinical evaluation and histopathologic examination as described [6]. All affected dogs had clinically normal parents and more than one puppy per litter was affected in many litters. Both sexes were affected (18 males and 22 females). These are features characteristic of an autosomal recessive disorder.

**Fig 2:**
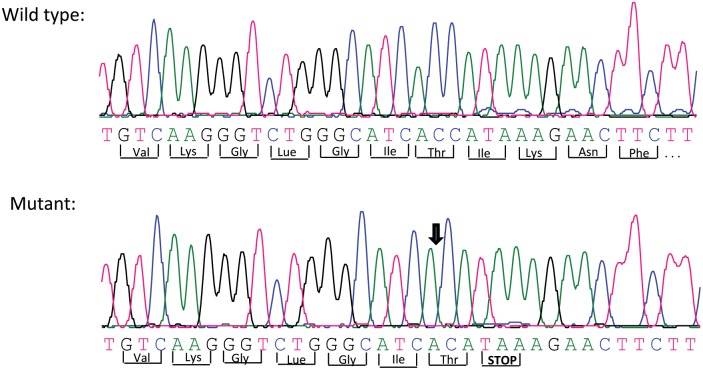
Sequencing chromatograms of a region within exon 6 of canine *NIPAL4*. Comparison of the sequences from the normal dog (upper panel) and from the dog affected with ARCI (lower panel) show a missing cytosine (arrow) in the affected dog.

The ARCI-affected dogs’ *NIPAL4* exon sequences were compared to the predicted exons based on the published canine genome sequence (see Materials and Methods) and to the sequencing results of family members and an unrelated clinically healthy dog. Sequencing revealed the deletion of the 157^th^ base of exon 6, a cytosine residue at the third position of a threonine codon, causing a translation reading frameshift resulting in a premature stop codon at codon position 249 (Fig 2). This deleted cytosine residue corresponds to CanFam3.1 canine reference genome sequence NC_06586.3, g.52737279del (residue 52,737,279 on canine chromosome CFA4).

### Restriction fragment length polymorphism and real-time PCR based assays

**Fig 3:**
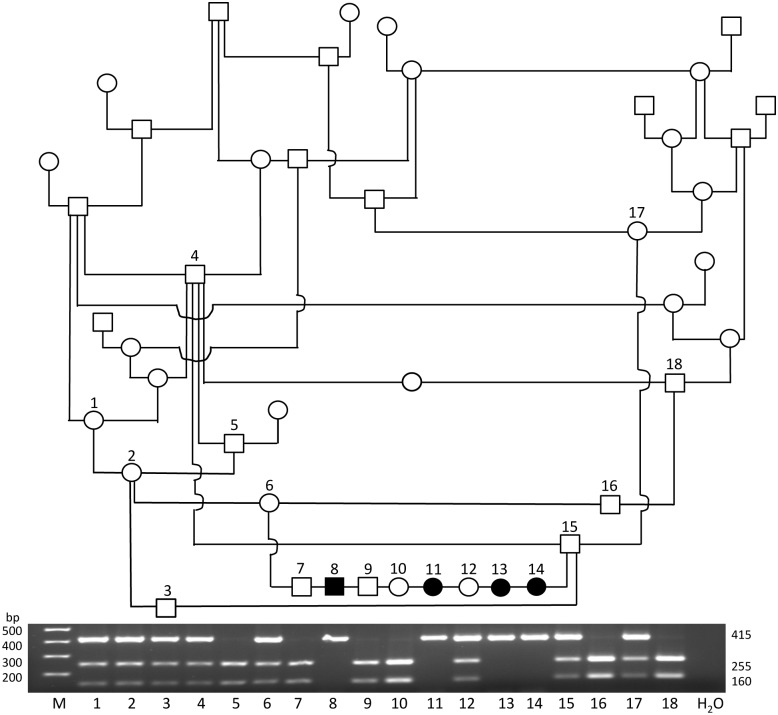
PCR-RFLP based genotyping results and corresponding pedigree of an ABD family with affected ARCI dogs. Four clinically affected dogs (8, 11, 13, and 14) were homozygous for the variant allele, their clinically healthy sire (6), dam (15) and a littermate (12) were heterozygous, and 3 littermates were homozygous for the wild type allele (7, 9, and 10). (M = marker, squares = males, circles = females, open symbols = clinically normal, filled in symbols = clinically affected). The p value for the association of homozygosity for the variant allele with the clinical phenotype (affected versus non-affected) is 0.0003 by the Fisher Exact Probability Test.

An RFLP based PCR assay was performed using samples (N = 18) from an extended ABD family that had produced ARCI-affected puppies. All animals in the pedigree (Fig 3) that were homozygous for the mutant allele were clinically and histologically affected with ARCI. Their clinically healthy sire and dam, along with several phenotypically normal family members, were heterozygous for the variant allele. The parents of the affected animals were heterozygous, which is consistent with an autosomal recessive mode of inheritance (Fig 3).

### Real time PCR based allelic discrimination assay

In order to rapidly and precisely detect the variant among the larger ABD population, a real time PCR-based allelic discrimination genotyping assay was performed in ABD dogs (N = 800) from North America, Australia, and Europe collected from 2008–2013. Among them, all 40 clinically diagnosed ARCI-affected dogs were homozygous for the single base deletion in exon 6. Of the 760 phenotypically normal dogs that had no clinical signs of skin disease, 274 (including 21 obligate carriers) were heterozygous for the variant and wild type allele, and constitute 34.3% of clinically healthy dogs.

## Discussion

This paper characterizes a deleterious molecular defect in the *NIPAL4* gene encoding ichthyin associated with ARCI in the ABD breed of dogs. A homozygous cytosine deletion in exon 6 of *NIPAL4* was identified in all 40 ABDs affected with ARCI in this study. Obligate carriers (N = 21) were confirmed heterozygous for this variant. DNA samples from 800 ABDs were collected from the North America, Europe and Australia during 2008–2013 and genotyped. While the population tested was not a random population, and probably biased to inflate the incidence of the disease-associated allele, the allele frequency of this single base deletion was 22.4%, with 39.3% of all dogs tested being either carriers or affected. The bias can be explained by assuming that owners of relatives of known affected dogs were more likely to test their dogs than owners that were not aware of affected relatives of their dogs. Since the introduction of the *NIPAL4* variant test, fewer numbers of affected dogs have been reported (MLC, unpublished data). It has allowed the breeders to retain dogs for breeding that have outstanding breed related qualities but are heterozygotes. Therefore, they can be mated with non-carriers without the fear of producing affected puppies while preserving desirable genes and thus, traits.

The canine *NIPAL4* gene resides on canine chromosome 4 and is composed of 6 exons that encode the ichthyin protein. While the human ichthyin is 466 amino acid residues in length (www.uniprot.org/uniprot/Q0D2K0), the canine protein is predicted to be composed of 404 amino acids (see Materials and Methods). The 62 additional amino acids in the human protein are at the amino terminus, due to the upstream location of a predicted in-frame methionine used as the start of translation in human, but absent in the dog. Indeed, Levere et al. [9] describe the human protein to be composed of 404 amino acid residues suggesting that the predicted start sequence in canine *NIPAL4* is orthologous to that in man. The altered reading frame in affected dogs maintains a threonine codon at position 248 but is immediately followed by a TAA stop codon, resulting in a truncated protein of only 248 amino acids. This truncated protein retains only 6 of the 9 predicted transmembrane domains of the mature protein in mice and humans, respectively [2, 9] (see Materials and Methods).

The *NIPAL4* gene encodes ichthyin, a putative transmembrane protein, which is expressed in the granular layer of the epidermis. This protein, with nine predicted transmembrane domains, is thought to be a Mg^2+^ transporter and plays a role in lipid metabolism during the epidermal development, but specific understanding of its function remains unknown [2, 4, 9]. To date, ten unique variants in NIPAL4 have been associated with various forms of ichthyosis in human patients, consisting of seven missense variants in exons 4, 5, and 6 [9–11], one nonsense variant in exon 2 [9], and two consensus splice site variants affecting splicing of introns 2 and 5 [10]. The nonsense and splice site variations seen in human patients, all of which are predicted to cause the production of a truncated protein, are most likely to cause a similar phenotype to the frameshift variant of the ABD. However, it is difficult to make direct comparisons for the two splice variants because they are each found as a single allele in patients whose second allele is c.527A>C/p.Ala176Asp [10]. In the case of the nonsense mutation, the protein is truncated at the equivalent of amino acid 83 of the canine ichthyin protein, predicting a much shorter protein.

Nonsyndromic ARCI has been reported in a number of other dog breeds with the molecular defect elucidated in several [6, 12–17]. In both humans and dogs, ARCI can be further classified as epidermolytic and nonepidermolytic based on light microscopy. Epidermolytic ichthyosis (aka epidermolytic hyperkeratosis) is specifically correlated with mutations in epidermal keratins [18]. In dogs, this disorder has been well characterized only in the Norfolk terrier (keratin 10, *KRT10*) [12]. In addition to ABDs, nonepidermolytic ARCI occurs in the Jack Russell terrier due to a defective structural protein (transglutaminase 1, *TGM1*) [13] and in great Danes due to a defect in a fatty acid transporter protein (*FATP4* aka *SLC27A4*) [19, 20]. A relatively common form of ARCI affects golden retrievers and, similar to ABDs, it is associated with a gene involved in epidermal lipid metabolism (phospholipase gene, *PNPLA1*) [14]. The phenotype in the ABD has some features that overlap those seen in the golden retriever (generalized white scale). As compared to the golden retriever, the ABD has 1) more severe generalized scaling with *Malassezia* overgrowth, 2) consistent clinical onset at birth, and 3) the presence of erythematous glabrous skin of the abdomen with adherent light brown scale [6, 14, 17]. In contrast to the ABDs, the golden retrievers also develop abdominal hyperpigmentation.

Humans with NIPAL4 deficient ARCI may be characterized phenotypically as non-bulbous congenital ichthyosiform erythroderma (CIE; fine white scale with erythema) or lamellar ichthyosis (LI; thick brown scale) as is typical for nonepidermolytic ARCI regardless of the genetic variant. The phenotype does not predict mutational status and can shift with age and therapies [5, 18]. Comparatively, the canine phenotype overlaps CIE and LI but remains constant among all dogs and throughout their lifespan. A feature of NIPAL4 deficient ARCI in humans is yellow palmar plantar keratoderma [21], and interestingly, the ABDs also develop paw pad hyperkeratosis with yellow discoloration, but it is not observed until over one year of age (EAM, unpublished observation) [6].

ARCI in American bulldogs is a phenotypically consistent disease, and the results of our study are highly suggestive that the disease is caused by nonfunctional truncated ichthyin protein. Ichthyin is clearly critical to the formation of a normal stratum corneum—the skin barrier. The clinical phenotype is the product of the epidermis’ attempt at mending the defective barrier and protecting the body from the ambient environment. Further investigation of this spontaneous large animal model are underway and may provide insight into the role of ichthyin in the formation of the lipid skin barrier and help us understand how to aid the epidermis in overcoming or bypassing the defect.
